# Supplementary material for: Primary and secondary anti-viral response captured by the dynamics and phenotype of individual T cell clones
Source: eLife. 2020 Feb 21;9:e53704. doi: 10.7554/eLife.53704 (PMC7060039; doi:10.7554/eLife.53704)
Supplement: Transparent reporting form [file elife-53704-transrepform.docx]

***eLife’s* transparent reporting form**

We encourage authors to provide detailed information *within their submission* to facilitate the interpretation and replication of experiments. If you have any questions, please contact us: [editorial@elifesciences.org](mailto:editorial@elifesciences.org).

**Sample-size estimation**

- You should state whether an appropriate sample size was computed when the study was being designed
- You should state the statistical method of sample size computation and any required assumptions
- If no explicit power analysis was used, you should describe how you decided what sample (replicate) size (number) to use

Please outline where this information can be found within the submission (e.g., page numbers or figure legends), or explain why this information doesn’t apply to your submission:

Current research is focused on deep sequencing of individual TCR alpha and beta repertoires after yellow fever vaccination, rather than measuring a single statistic for large groups of donors. The timepoints for blood samples collection were selected to capture immune repertoire status before the response (day 0), during the acute stage of the response (days 5-21) and at memory stage (day 45) based on multiple previous studies [Miller et al. 2008, Akondy et al. 2009, Blom et. al 2013, Pogorelyy et al. 2018]. All sequence library information (cell source, library preparation method), as well as sample sizes (approximate number of cells, the total number of reads, number of unique molecular identifiers, and number of unique clonotypes), are presented in Figure 1 – source data 1. The number of reads per library was chosen to be at least two times more than the number of unique RNA molecules, as it allows us to use advanced PCR error-correction techniques [Shugay et al. 2014].

**Replicates**

- You should report how often each experiment was performed
- You should include a definition of biological versus technical replication
- The data obtained should be provided and sufficient information should be provided to indicate the number of independent biological and/or technical replicates
- If you encountered any outliers, you should describe how these were handled
- Criteria for exclusion/inclusion of data should be clearly stated
- High-throughput sequence data should be uploaded before submission, with a private link for reviewers provided (these are available from both GEO and ArrayExpress)

Please outline where this information can be found within the submission (e.g., page numbers or figure legends), or explain why this information doesn’t apply to your submission:

For the bulk PBMC samples on all timepoints after yellow fever vaccination, we used two biological replicates (Figure 1-source data 1). For this, we collected two portions of blood and proceed through all library preparation steps (PBMC isolation, cDNA synthesis, and PCR amplification) independently. These biological replicates were used for noise estimation during YF-responding clones’ identification with edgeR, as described in detail in the Methods section. All other sequencing experiments were performed once (no technical replicates). The list of all samples can be found in Figure 1 – source data 1. We did not exclude any datasets from the analysis. Raw data is in the Short Read Archive under accession number PRJNA577794.

Raw TCR sequencing data were analyzed with MIGEC and MIXCR software, as described in Methods section page 13.

Raw data for single-cell RNAseq was preprocessed with Cell Ranger 2.2.0 software with default parameters, and GRCh38-1.2.0 reference genome was used for the gene alignment. Seurat 3.0 software was then used for the analysis, as described in Methods page 14.

Single-cell TCR profiling data was processed with Cell Ranger 2.2.0 with default parameters. Only clonotypes that had a single alpha and single beta TCR chains and were present more than two times in a data were taken for further analysis. The resulting list of paired alpha-beta clonotypes from 10x Genomics experiment could be found in Figure 4-source data 2.

**Statistical reporting**

- Statistical analysis methods should be described and justified
- Raw data should be presented in figures whenever informative to do so (typically when N per group is less than 10)
- For each experiment, you should identify the statistical tests used, exact values of N, definitions of center, methods of multiple test correction, and dispersion and precision measures (e.g., mean, median, SD, SEM, confidence intervals; and, for the major substantive results, a measure of effect size (e.g., Pearson's r, Cohen's d)
- Report exact p-values wherever possible alongside the summary statistics and 95% confidence intervals. These should be reported for all key questions and not only when the p-value is less than 0.05.

1) On page 2 we report edgeR FDR adjusted p-value <0.01 (quantile-adjusted conditional likelihood test implemented by edgeR package), see “Repertoire data analysis” section in Methods for details
2) On page 5 we report Pearson correlation coefficient for clonotype concentrations 45 days after the primary vaccination and concentrations ~540 days after before the booster vaccination. Pearson correlation: r=0.46, p<0.0001, N=1580. Data is shown in Figure 2-figure supplement 2.
3) In Fig 3 legend, we report exact p-values for Mann Whitney U-test for YF-clone concentrations in effector memory (EM) and terminally differentiated effector memory (EMRA) on days 15 and 45 after immunization.
4) We used the exact Fisher test with Benjamini-Hochberg multiple testing correction to find V and J segments significantly enriched in the repertoire of NS4B-specific TCRs (see Fig 4 legend, page 6).
5) In Fig 5 legend, we report chi-squared test with Monte-Carlo estimated p-value to test if the distribution of clonotypes between cell phenotype clusters is not random (page 9).
6) In Figure 4-figure supplement 4A legend, we report p-values for Mann Whitney U-test for the mean number of contacts between CDRs of TCRs of different motif groups and immunodominant yellow fever peptide.
7) In Figure 5-figure supplement 2B legend, we report p-values for Mann Whitney U-test for the clonal concentrations of different clonotypes after yellow fever immunization.
8) Differential gene expression analysis for single-cell data is described in “Single-cell gene expression analysis” Methods section. Exact and adjusted p-values for all genes differentially expressed between clusters of cells and TCR clonotypes are provided in Figure 5-source data 1 and Figure5-source data 2 respectively.

Please outline where this information can be found within the submission (e.g., page numbers or figure legends), or explain why this information doesn’t apply to your submission:

(For large datasets, or papers with a very large number of statistical tests, you may upload a single table file with tests, Ns, etc., with reference to page numbers in the manuscript.)

**Additional data files (“source data”)**

- We encourage you to upload relevant additional data files, such as numerical data that are represented as a graph in a figure, or as a summary table
- Where provided, these should be in the most useful format, and they can be uploaded as “Source data” files linked to a main figure or table
- Include model definition files including the full list of parameters used
- Include code used for data analysis (e.g., R, MatLab)
- Avoid stating that data files are “available upon request”

Please indicate the figures or tables for which source data files have been provided:

Figure 1-source data 2 contains source data for Figure 1B.

Figure 1-source data 3 contains lists of all YF-responding clonotypes identified by EdgeR software as described in detail on page 13. These clonotypes were used for Figures: Figure 1C, Figure 1D, Figure 2, Figure 4A, Fig. 1-suppl. 1, Fig. 2-suppl. 1, Fig. 2-suppl. 2, Fig. 2-suppl. 3, Fig. 4-suppl. 2

Figure 2-source data 1,2 contain raw data for Figure 2 and Fig. 2-suppl. 1.

Figure 3-source data 1 contains raw data for Figure 3A.

Figure 3-source data 2 contains raw data for Figure 3B and Fig. 3-suppl. 2A,C.

Figure 3-source data 3 contains raw data for Figure 3C and Fig. 3-suppl. 2B,D.

Figure 4-source data1 contains source data for similarity network plots of CDR amino acid regions of NS4B-specific TCRs (Figure 4B, 4C) and histograms of CDR3 amino acid lengths of NS4B-specific TCRs (Figure 4D, E).

Figure 4-source data 2 contains data for the joint similarity network of NS4B-specific alpha-beta TCRs (Figure 4F), alluvial plots for TRAV-TRBV co-occurrence (Figure 4G, Fig. 4-suppl. 3A, C), Figure 5C, Fig5-suppl2B as well as the data used for TCR-pMHC modeling (see Methods, page 14) and TCRdist analysis (Fig. 4- suppl. 3B).

Figure 5-source data 1 contains list of differentially expressed genes between NS4B-specific cells. Figure 5-source data 1 was used for Figure 5B, Fig. 5-suppl. 1.

Figure 5-source data 2 contains list of differentially expressed genes between NS4B-specific clonotypes. Figure 5-source data 2 was used for Fig. 5-suppl. 2.
